# Promoting the Positive Experience of Caregiving: A Systematic Review of the Positive Aspects of Caregiving Scale

**DOI:** 10.1093/geroni/igaa057.107

**Published:** 2020-12-16

**Authors:** Yeonjung Lee, Lun Li

**Affiliations:** 1 University of Calgary, Calgary, Alberta, Canada; 2 Simon Fraser University, Vancouver, British Columbia, Canada

## Abstract

While caregivers endure considerable challenges, including potentially deleterious effects on their own well-being, the possibility of positive outcomes for caregivers, such as feeling rewarded and satisfied, has been recognized in scholarly research as well. Caregiving outcomes can be complicated by different aspects of caregiving experiences. Particularly, the Positive Aspect of Caregiving (PAC) scale covers four main positive aspects including caregiving personal gains, motivation for caregiving role, caregiver satisfaction, as well as self-esteem and social aspect of caring. The objective of this study is to summarize the research findings based on the PAC scale and further improve the understanding of the positive experience perceived by caregivers. A systematic literature review was conducted to identify the existing evidences. This systematic review identified empirical research studies written in English focusing on a PAC scale that were published in a peer-reviewed journal between 2004 and 2018. Systematic search was carried out within 10 databases. After careful review of 193 abstracts yielded from the databases, 38 journal articles were identified to have used the PAC scale. Analysis of the selected literature provides four themes that how PAC has been used. The themes are to measure caregiving outcomes, test the effect of caregiver program intervention, complement other caregiving outcome scales, and test the validity in other languages and countries. This systematic review highlights the importance of taking into account of the positive experience of caregiving and further to promote it in a way of buffering the negative outcomes of caregiving.

